# Dental Surgery

**Published:** 1895-05

**Authors:** 


### Editorial, Etc.

DENTAL SURGERY.—At the meeting of the Odontological Society of Pennsylvania Saturday night Dr. B. Holly Smith, of Baltimore, read a paper in favor of continuing the present plan of a three-years' course in the dental schools and not prolonging it to four years. Dr. William Shaw Twilley and Dr. Richard Grady, also of Baltimore, contributed historical sketches respecting the protest of Baltimore dentists against the classification of dentists as manufacturers, which subject is now in the care of the American Dental Association. They argued that time and success had vindicated the protest, because the census bureau had discontinued its efforts; that as far back as August, 1890, in the public press of Baltimore the claim was made that everything which dentists make in practice is surgical; that filling teeth is a surgical operation; that, the exhibits at the International Exhibition in Philadelphia in 1876, embraced pivot teeth, dentures, apparatus for the correction of congenital malformations of the palate and loss by scrofulous and syphilitic necrosis were surgical on the testimony of the judges, all of whom were in medicine and surgery: that in September, 1890, they had challenged the definition of mechanical dentist as given by the census office which was to "embrace all branches of the profession except the extraction of teeth" and the term was afterward given a new meaning; that they had never gotten an answer to the query, "why the repair of the teeth should come under mechanical employment and the repair of the body belong to a liberal profession." The subject was further discussed by Dr. L. Ashley Faught, who conducted the correspondence for the Philadelphia dentists with the census bureau and by Dr. J. D. Thomas, who went from Philadelphia to Washington to defeat the bill which provided fine and imprisonment for refusal to answer questions.—*From the Phila. letter of the Baltimore Sun, March 10th, 1895.*

